# A Multi-Criteria Decision Aid Tool for Radiopharmaceutical Selection in Tau PET Imaging

**DOI:** 10.3390/pharmaceutics15041304

**Published:** 2023-04-21

**Authors:** Ilker Ozsahin, Efe Precious Onakpojeruo, Berna Uzun, Dilber Uzun Ozsahin, Tracy A. Butler

**Affiliations:** 1 Brain Health Imaging Institute, Department of Radiology, Weill Cornell Medicine, New York, NY 10065, USA; 2 Operational Research Center in Healthcare, Near East University, Nicosia 99138, TRNC, Turkey; 3 Department of Statistics, Carlos III University of Madrid, Getafe, 28903 Madrid, Spain; 4 Medical Diagnostic Imaging Department, College of Health Sciences & Research Institute for Medical and Health Sciences, University of Sharjah, Sharjah P.O. Box 26666, United Arab Emirates

**Keywords:** positron emission tomography, radiopharmaceuticals, tauopathies, Alzheimer’s disease, decision analysis, fuzzy PROMETHEE

## Abstract

The accumulation of pathologically misfolded tau is a feature shared by a group of neurodegenerative disorders collectively referred to as tauopathies. Alzheimer’s disease (AD) is the most prevalent of these tauopathies. Immunohistochemical evaluation allows neuropathologists to visualize paired-helical filaments (PHFs)—tau pathological lesions, but this is possible only after death and only shows tau in the portion of brain sampled. Positron emission tomography (PET) imaging allows both the quantitative and qualitative analysis of pathology over the whole brain of a living subject. The ability to detect and quantify tau pathology in vivo using PET can aid in the early diagnosis of AD, provide a way to monitor disease progression, and determine the effectiveness of therapeutic interventions aimed at reducing tau pathology. Several tau-specific PET radiotracers are now available for research purposes, and one is approved for clinical use. This study aims to analyze, compare, and rank currently available tau PET radiotracers using the fuzzy preference ranking organization method for enrichment of evaluations (PROMETHEE), which is a multi-criteria decision-making (MCDM) tool. The evaluation is based on relatively weighted criteria, such as specificity, target binding affinity, brain uptake, brain penetration, and rates of adverse reactions. Based on the selected criteria and assigned weights, this study shows that a second-generation tau tracer, [^18^F]RO-948, may be the most favorable. This flexible method can be extended and updated to include new tracers, additional criteria, and modified weights to help researchers and clinicians select the optimal tau PET tracer for specific purposes. Additional work is needed to confirm these results, including a systematic approach to defining and weighting criteria and clinical validation of tracers in different diseases and patient populations.

## 1. Introduction

Neurodegenerative disorders, such as Alzheimer’s disease (AD), are characterized by progressive neuron loss and brain function impairment [1,2]. The inappropriate buildup of aggregates of the proteins amyloid and tau in the brain is a key contributor to AD [3]. Once only visible after death via neuropathological examination, amyloid and tau can now be visualized in vivo using positron emission tomography (PET). Amyloid PET tracers have been available for almost two decades. More recently, researchers have been developing tau-specific PET tracers to measure tau deposition in AD and other disorders and to track tau spread over time [1,2,4,5]. Several tau PET tracers are now being used for human research, and one is approved for clinical use [3,5,6]. The ability to detect and quantify tau pathology in vivo using these tracers can aid in the early diagnosis of AD and other tauopathies and provide a way to monitor disease progression and determine the effectiveness of therapeutic interventions [7]. However, more studies and evaluations are needed to fully understand the value of different tau PET radiotracers as biomarkers, including how to choose the appropriate tracer for particular research or clinical purposes [4,8].

In this study, tau PET tracers were evaluated, compared, and ranked using a multi-criteria decision-making (MCDM) tool based on human knowledge. MCDM methods allow for the evaluation and comparison of available options based on selected criteria and assigned relative weighting. MCDM has become an essential tool for decision makers in various fields, such as business, engineering, healthcare, and public policy [9,10,11]. Here, we applied MCDM methods to the selection of the optimal radiopharmaceutical for tau PET imaging to aid in the diagnosis and disease monitoring of tauopathies such as AD. 

One of the most widely used MCDM methods is the fuzzy preference ranking organization method for enrichment of evaluations (PROMETHEE). PROMETHEE was first introduced in the early 1980s by Brans and Vincke as a tool for solving decision problems with multiple criteria [12,13]. The basic idea behind PROMETHEE is to rank alternatives based on pairwise comparison using the preference functions. The preference values can be calculated using one of the preference functions that captures the preferences of the decision-maker for each criteria. PROMETHEE can handle both quantitative and qualitative data, making it a versatile and flexible method compared to other MCDM methods [9,10,11,14,15]. Similarly, PROMETHEE aims to enrich decision making by providing a systematic and transparent approach to handling multiple criteria. Over the years, PROMETHEE has been applied in a wide range of contexts, from environmental management to financial analysis, and recently in medicine [9,10,11,14,15]. In addition to the original PROMETHEE method, several variations and extensions have been developed to address specific decision problems. These include PROMETHEE II, which accounts for imprecise information, PROMETHEE III, which considers interdependence among criteria, and PROMETHEE IV, which incorporates group decision making [16]. 

The fuzzy PROMETHEE tool was deployed in the present study. We also compared our PROMETHEE results with two other commonly used methods: weighted sum method and technique for order preference by similarity to ideal solution (TOPSIS). Although the fuzzy PROMETHEE method has been used in several biomedical and other contexts [14,15], to the best of our knowledge neither the fuzzy PROMETHEE nor any MCDM method has been proposed for use in prioritizing promising and effective PET tracers. The purpose of this study was to provide researchers and clinicians with an example of how MCDM methods of evaluation and comparison can be used to select the optimal tau PET tracer based on defined criteria.

The sections of this study are summarized as follows: Section 2 presents the background of tau PET imaging. This section explains the PET imaging technique and how it is used in combination with other structural imaging techniques to study biochemical processes in vivo. The first and second generations of tau PET radiotracers are also discussed. Section 3 focuses on the methodology used in this research, which was the MCDM approach. Section 4 shows the results of the study and the result validation processes. Section 5 discusses the results and limitations. Finally, Section 6 concludes the study with recommendations.

## 2. Background on Tau PET Imaging

PET is a noninvasive imaging technique that can be used to gain insight into biochemical processes occurring in the brain and body of living subjects. This type of nuclear imaging makes use of a bioactive molecule that has been modified to contain a radioisotope that emits positrons. When the bioactive molecule interacts with its target in tissue, the decay of the position over the course of time can be detected [8]. This collection of decays, broken down by time and location, allows scientists to localize and study biochemical processes in vivo. To localize this dynamic information with greater precision, PET is often used with a structural technique such as CT or MR imaging. The most prevalent PET isotopes are carbon-11 (^11^C), fluorine-18 (^18^F), nitrogen-13 (^13^N), oxygen-15 (^15^O), and gallium-68 (^68^G). ^18^F is the most commonly used radioactive element in PET imaging due to the fact of its availability and practicality. On the other hand, ^11^C has excellent chemical properties for PET imaging, but its short half-life and requirement for onsite synthesis poses logistical challenges, making it not as widely used as ^18^F [5]. In PET imaging, the distribution of the radiotracer can provide both quantitative and qualitative information. Quantitatively, the amount of radiotracer uptake in a specific tissue or organ can be measured and analyzed, providing information on the function and metabolism of that tissue or organ. This is important for assessing changes in metabolism that may be indicative of disease or other medical conditions. Qualitatively, PET imaging can be interpreted by a radiologist to assess the location and extent of abnormalities, such as tumors or inflammation, in the body. This is conducted by visually comparing the distribution of the radiotracer in the abnormal tissue to that of surrounding healthy tissue. PET imaging is a valuable medical and research tool that allows for both quantitative and qualitative analysis, providing a comprehensive view of the function and structure of tissues and organs.

PET has proven extremely useful in understanding neurodegenerative diseases such as Alzheimer’s disease (AD). PET tracers for the AD protein amyloid-B (AB) have been available for approximately two decades. PET tracers for tau were developed more recently. Importantly, the pattern of tau deposition and spread detected with tau PET correlates strongly with cognitive impairment and AD progression; this is not the case for AB PET. Amyloid and tau PET imaging are typically combined with information from other diagnostic tools, such as cerebrospinal fluid analysis, MRI, and cognitive and clinical assessments. By combining these complementary measures, clinicians and researchers can obtain a more complete understanding of the disease state and monitor disease progression over time. 

### Tau PET Radiotracers

Tau PET radiotracers bind to tau in paired-helical filaments in the brain, allowing tau lesions to be visualized and quantified using positron emission tomography (PET) imaging. These tracers have the potential to aid in the early diagnosis and monitoring of neurodegenerative diseases, such as AD and other tauopathies [5]. These tracers are designed to bind specifically to tau protein and not to other proteins such as AB. Currently, most tau radiotracers are sensitive only to tau in AD, which consists of a mixture of 3R and 4R tau. Effort is underway to identify tracers to detect tau in other tauopathies, such as chronic traumatic encephalopathy (CTE), progressive supranuclear palsy (PSP), and Pick’s disease. One tau PET radiotracer has been approved for clinical use in the United States: [^18^F]AV-1451 ([^18^F]Flortaucipir, [^18^F]T807, and Tauvid). Other tracers used in this study included first-generation [^11^C]PBB3, [^18^F]THK5105, [^18^F]THK5117, [^18^F]THK5317, and [^18^F]THK5351 and second-generation [^18^F]MK-6240, [^18^F]GTP1 (Genentech Tau Probe 1), [^18^F]PM-PBB3 ([^18^F]Florzolotau and [^18^F]APN-1607), [^18^F]JNJ067 ([^18^F]JNJ-64326067), [^18^F]JNJ-311 ([^18^F]JNJ-64349311), [^11^C]RO-643 ([^11^C]RO-6931643), [^11^C]RO-963 ([^11^C]RO-6924963), [^18^F]RO-948 ([^18^F]RO69558948), and [^18^F]PI-2620, which are experimental [7,17,18,19,20,21,22,23,24,25,26]. Tau radiotracers can be categorized as first or second generation. Most first-generation tracers were developed to bind to the N-terminal or mid-region of the tau protein, which is known to be a major site of aggregation in these diseases [6]. These first-generation tau PET radiotracers are able to detect tau pathology in the brain, but they have some limitations, including high levels of nonspecific binding, in particular to the enzyme monoamine oxidase B (MAOB). Second-generation tau PET radiotracers have been developed to address some of the limitations of first-generation tau PET radiotracers [3]. They have been designed to be more selective, with higher binding affinities, and to bind to different regions of the tau protein, such as the C-terminal and the microtubule-binding domain [21]. Both first- and second-generation tau PET radiotracers have been used in several studies (e.g., [27,28,29,30,31,32]) to evaluate patients with AD and other tauopathies. These studies have shown that these tracers can accurately detect tau pathology in the brain and can provide valuable information about the distribution and progression of tau pathology in neurodegeneration. However, it is important to note that these tracers are still in the early stages of development, and more research is needed to fully understand their potential clinical applications. Here, we applied MCDM methods to evaluate and compare first- and second-generation tau PET radiotracers.

## 3. Methodology

Multi-criteria methods are decision-making tools that allow decision-makers to evaluate and compare multiple criteria or alternatives simultaneously [11]. These methods are widely used in various fields, including the medical field, to support complex decision-making processes that involve multiple objectives, stakeholders, and uncertain factors [14]. In the field of artificial intelligence, it is a potent tool with enormous potential, as it addresses the question of how to evaluate competing choices according to multiple criteria. One of the applications of multi-criteria methods in the medical field is in healthcare technology assessment, which is the systematic evaluation of medical devices, drugs, and procedures to determine their safety, effectiveness, and cost-effectiveness. MCDM is a common approach used in healthcare technology assessment to support the selection of the most appropriate healthcare technology by considering multiple criteria, such as clinical efficacy, safety, patient preferences, and economic factors. MCDM can help decision-makers to make informed and transparent decisions that consider a broad range of perspectives and trade-offs [9,10,11]. 

Another application of multi-criteria methods in the medical field is in clinical decision making, where the use of decision support systems can help physicians make informed decisions that consider multiple criteria, such as patient characteristics, medical history, clinical guidelines, and treatment options. MCDM can be used in decision support systems to evaluate the relative importance of different criteria and to provide personalized recommendations based on the patient’s specific needs and preferences [14,15]. Multi-criteria methods can also be used in healthcare resource allocation, where the goal is to allocate limited resources, such as in healthcare budgets, to different healthcare programs or interventions. MCDM can help decision-makers to prioritize and allocate resources based on multiple criteria, such as health outcomes, costs, equity, and feasibility [9,10,11]. The use of multi-criteria methods in the medical field can help improve decision-making processes, enhance transparency and accountability, and promote the efficient use of healthcare resources. We propose using MCDM methods to compare and evaluate the different tau PET tracers and their usefulness. The goal is for researchers, clinicians, and other decision-makers to have easier access to the information they need to make sound choices when selecting radiopharmaceuticals for use in tau PET. 

### 3.1. Application of Fuzzy PROMETHEE

As an approach to the implementation of MCDM, the use of fuzzy PROMETHEE is encouraged because it is sensitive and applicable when vague information occurs in the decision environment in which it is to be implemented. It is well known for its effectiveness in providing decision-makers with more options to consider various forms of uncertainty based on available criteria, and it can be applied to real-world problem structures. Both PROMETHEE I (a partial ranking structure) and PROMETHEE II are presented in detail in a study in [10]. Fuzzy logic allows for the proper analysis of ambiguous data before decisions are made. Fuzzy PROMETHEE is a hybrid model that relies on the assessment of uncertain instances; it finds widespread use in many fields of study, including science, technology, engineering, medicine, and sociology [33]. Two pieces of data are necessary for the PROMETHEE technique to work effectively: preference functions and the importance weight of the criteria [34,35]. In the PROMETHEE approach, six distinct preference functions are available for the determination of the preference of alternatives compared to others for each criteria: normal, U-shaped, V-shaped, level, linear, and Gaussian preference functions [36,37].

To evaluate the tau PET tracers, this study proposed several criteria and assigned weights to each criteria based on available expert opinions. The aforementioned criteria were optimized for use with fuzzy PROMETHEE by employing a triangular linguistic fuzzy scale, as shown in Table 1. In addition, the fuzzy values were defuzzified using the Yager index, which is calculated as (3N − a + b)/3, where N is the center of the set, a is the distance between the center and left bound, and b is the distance between the center and the right bound. The Yager index is a recommended method of defuzzification because it considers all of the assigned points in the set for defuzzification. 

There are 5 main steps in the PROMETHEE method to be applied for the MCDM analysis:

- The preference function P_j_(d) of each criteria j should be defined;
- The importance weights of each criteria w_t_ = (w_1_, w_2_, …, w_k_) should be defined;
- For each of the alternative pairs $a_{t}$, $a_{t^{\prime }}$ ∈ A, the outranking relation (π) should be determined by:

(1) $$\pi \left(a_{t},a_{t^{\prime }}\right)=\sum_{k=1}^{K}w_{k}.\left[p_{k}\left(f_{k}\left(a_{t}\right)-f_{k}\left(a_{t^{\prime }}\right)\right)\right],\;\;AXA\to \left[0,1\right]$$

where π (a, b) denotes the preference indices, which shows the preference intensity for an alternative $a_{t}$ in comparison to an alternative $a_{t^{\prime }}$ while counting all criteria.

- The positive and negative outranking flows should be determined as follows:

A positive outranking flow of the alternative $a_{t}$:

(2) $$\Phi^{+}\left(a_{t}\right)=\frac{1}{n-1}\sum_{\begin{array}{c} t^{\prime }=1\\ t^{\prime }\neq t \end{array}}^{n}\pi \left(a_{t},a_{t^{\prime }}\right)$$

A negative outranking flow of the alternative $a_{t}$:

(3) $$\Phi^{-}\left(a_{t}\right)=\frac{1}{n-1}\sum_{\begin{array}{c} t^{\prime }=1\\ t^{\prime }\neq t \end{array}}^{n}\pi \left(a_{t^{\prime }},a_{t}\right)$$

where n denotes the number of alternatives; $\Phi^{+}\left(a_{t}\right)$ defines the strength of alternative $a_{t}$ ∈ A, while the negative outranking flow $\Phi^{-}\left(a_{t}\right)$ defines the weakness of alternative $a_{t}$ ∈ A.

PROMETHEE I determine the partial pre-order of the alternatives, while PROMETHEE II determines the net ranking to alternatives. The partial pre-order of the options can be determined based on the following statements.

Via PROMETHEE I, alternative $a_{t}$ is selected to alternative $a_{t^{\prime }}$ ($a_{t}Pa_{t^{\prime }})$ if it satisfies either of the statements given below.

(4) $$\left\{\begin{array}{c} \Phi^{+}\left(a_{t}\right)\geq \Phi^{+}\left(a_{t^{\prime }}\right)\;and\;\Phi^{-}\left(a_{t}\right)<\Phi^{-}\left(a_{t^{\prime }}\right)\\ \Phi^{+}\left(a_{t}\right)>\Phi^{+}\left(a_{t^{\prime }}\right)\;and\;\Phi^{-}\left(a_{t}\right)=\Phi^{-}\left(a_{t^{\prime }}\right) \end{array}\right.$$

$\mathrm{Alternative}\;a_{t}$ is indifferent to alternative $a_{t^{\prime }}$ ($a_{t}Ia_{t^{\prime }})$ if:

(5) $$\Phi^{+}\left(a_{t}\right)=\Phi^{+}\left(a_{t^{\prime }}\right)\;and\;\Phi^{-}\left(a_{t}\right)=\Phi^{-}\left(a_{t^{\prime }}\right)$$

and a_t_ is incomparable to a_t’_(a_t_ Ra_t’_) if:

(6) $$\left\{\begin{array}{c} \Phi^{+}\left(a_{t}\right)>\Phi^{+}\left(a_{t^{\prime }}\right)\;and\;\Phi^{-}\left(a_{t}\right)>\Phi^{-}\left(a_{t^{\prime }}\right)\\ \Phi^{+}\left(a_{t}\right)<\Phi^{+}\left(a_{t^{\prime }}\right)\;and\;\Phi^{-}\left(a_{t}\right)<\Phi^{-}\left(a_{t^{\prime }}\right) \end{array}\right.$$

- The net outranking flow can be calculated for each alternative using Equation (7):

(7) $$\Phi^{net}\left(a_{t}\right)=\Phi^{+}\left(a_{t}\right)-\Phi^{-}\left(a_{t}\right)$$

Via PROMETHEE II, the complete order with net flow can be determined as:

(8) $$a_{t}\;\mathrm{is}\;\mathrm{preferred}\;\mathrm{to}\;a_{t^{\prime }}\;(a_{t}Pa_{t^{\prime }})\;\mathrm{if}\;\Phi^{net}\left(a_{t}\right)>\Phi^{net}\left(a_{t^{\prime }}\right)$$

(9) $$a_{t}\;\mathrm{is}\;\mathrm{indifferent}\;\mathrm{to}\;a_{t^{\prime }}\;(a_{t}Ia_{t^{\prime }})\;\mathrm{if}\;\Phi^{net}\left(a_{t}\right)=\Phi^{net}\left(a_{t^{\prime }}\right)$$

The higher $\Phi^{net}\left(a_{t}\right)$ value provides the better alternative.

During the decision-making process, selected criteria are used to evaluate alternatives. Since not all criteria are of equal importance, weights must be assigned. This means that the criteria that are most important are given more weight, while those that are less important are given less. It is possible and expected that various decision-makers will use different criteria and have varying preferences for potential solutions. 

### 3.2. Comparison with the Weighted Sum Method and TOPSIS

For this study, we compared the results using PROMETHEE to those obtained using two other commonly used methods for multiple-criteria decision analysis: weighted sum method and TOPSIS method.

The weighted sum method is a simple and intuitive approach in which each criteria is assigned a weight, and the weighted scores for each alternative are summed to obtain a total score. The alternative with the highest total score is considered the most preferred.

The TOPSIS method is a technique that evaluates alternatives by comparing them to an ideal solution and a negative ideal solution. The ideal solution is the alternative that has the best performance for each criteria, while the negative ideal solution has the least performance for each criteria. The distances of each alternative from the ideal and negative ideal solution are calculated, and the alternative that has the shortest distance from the ideal solution and the longest distance from the negative ideal solution is considered the most preferred [10,11].

### 3.3. Defining Criteria

When evaluating tau PET tracers, relevant evaluation criteria for optimal visualization of brain tau aggregates include target binding affinity, selectivity for target protein, ability to penetrate blood–brain barrier, low nonspecific binding, radioactive metabolites, reversible versus nonreversible kinetics, and sensitivity to non-AD tau [38,39]. For this initial study and due to the limited information in the existing literature, this study focused only on the following criteria: target binding affinity, specificity, brain uptake/penetration, and adverse reactions. 

#### 3.3.1. Target Binding Affinity

A radiotracer must bind to its target (tau) with sufficient affinity at a low injection dose to allow it to be detected using PET. 

#### 3.3.2. Specificity

Specificity refers to the tracer having greater affinity to tau compared to other proteins in the brain. This is important in order to minimize off-target binding. 

#### 3.3.3. Brain Uptake and Penetration

This criterion reflects the amount of injected tracer that passes the blood–brain barrier and enters the brain and is available to interact with tau deposits in the brain. If a tau PET radiotracer has a poor brain penetration rate, it will not be able to detect brain tau pathology efficiently. This is a general requirement for all brain PET radiotracers.

#### 3.3.4. Adverse Reactions

PET tracers with high rates of adverse reactions, such as allergic reactions, headaches, or nausea, are inappropriate for clinical use. Adverse effects can vary depending on the specific radiotracer and the individual patient [40]. Therefore, it is important to carefully evaluate the safety profile of a tau PET radiotracer before it is used in a clinical setting to ensure that it is safe and well tolerated for patients [41]. The adverse effects of radiotracers are generally considered to be minimal, as they are administered at subpharmacologic doses and rapidly cleared after the imaging procedure.

In MCDM, the ratings of very high (VH), high (H), medium (M), low (L), and very low (VL) are commonly used to quantify the importance or weight of each criteria in the decision-making process [42,43]. The weight assignments are typically based on expert opinion and desired outcome. As shown in Table 1, in this study, we weighted specificity and brain uptake/penetration as VH, target binding affinity as H, and adverse reaction criteria as M. These weightings were assigned based on the available literature but should not be considered definitive, and they were chosen primarily to illustrate the methods. The assigned weights will differ based on the intended purpose. The weights of each criteria were used to calculate the overall rank of each alternative in the decision-making process. The higher the rating of a criteria, the more weight it carries in the overall decision.

## 4. Results

Table 2 below is the complete dataset used in this study.

Using fuzzy PROMETHEE to evaluate the selected criteria and by assigning weights and preference functions, it was determined that the [^18^F]RO-948 tau radiotracer came first in the ranking of tau PET tracers. This was accomplished with a net flow of 0.0051. The [^11^C]RO-643 has an outranking net flow of 0.0045, making it the second most preferred alternative. As can be seen in Table 3, [^18^F]THK5351, [^18^F]THK5317, [^18^F]THK5117, and [^18^F]THK5105 had the lowest outranking net flow of any of the tau PET tracers, which put them in the position of being the least preferred. However, it is important to note that if different weights are assigned to each of the selected criteria, the results of the outranking could be different.

**Figure 1:**
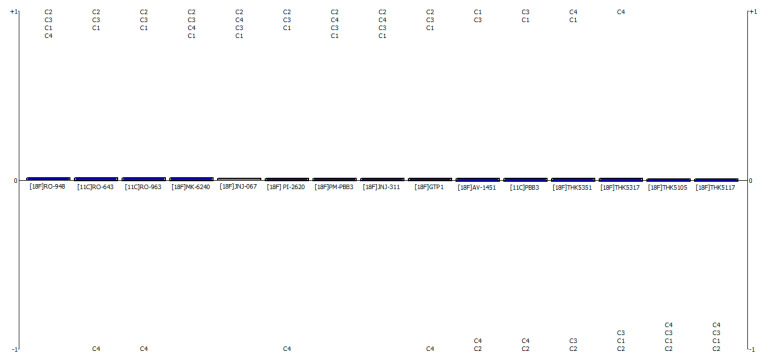
Positive and negative aspects of the alternatives. c1 is specificity, c2 is target binding affinity, c3 is brain uptake and penetration, and c4 is adverse reaction. The criteria that stand on the upper side of the plot are considered positive aspects, while the ones below zero mean that they exhibit undesired properties.

Tau PET radiotracers’ strengths and weaknesses, as well as their final ranking, are depicted in Figure 1. Every tau radiotracer that was considered for this analysis is ranked from the most preferred to the least preferred in this graph. The strengths of the alternatives are represented by criteria with values greater than 0, while weaknesses are indicated by values below 0. A diagram is used to show the overall process’s flow, with the various options listed from best to least on the horizontal axis. The criteria form a vertical bar to depict the alternatives available. Each segment of the bar illustrates how a different criterion impacts the net flow value of the alternatives. When a criterion is provided, its relative importance is shown as a weighted vertical bar whose height is proportional to the disparity between positive and negative preference flow. The indicators at the vertical bar’s extremes have the most extreme positive and negative values.

### 4.1. Sensitivity Analysis

To evaluate the efficacy of our proposed approach, we performed a sensitivity analysis in this section. The purpose of this sensitivity analysis was to determine the degree to which our previously generated result conforms to the proposed method. The sensitivity analysis investigates how changing the weights of the evaluation criteria affects the final order in which the tau PET radiotracers were ranked. Our goal in conducting this sensitivity analysis was to determine how varying the relative importance of the criteria we chose might affect the reliability of our definitive result. As part of the sensitivity analysis, we experimented with changing the weight of just one criterion slightly while maintaining all others unchanged. In the present investigation, importance weights were determined for each criterion on a linguistic scale (Table 1). As shown in Table 4, the weight of one crucial criterion—specificity, which was previously weighted as very high—was reduced to moderate.

Table 4 shows the weight ranges that can be changed without changing the final ranking that fuzzy PROMETHEE produced shown in Table 3. These weight ranges are articulated linguistically in Table 1. The specificity criterion had an initial weight of VH, but now it has a weight of H.

Based on the data presented in Table 5, we can infer that changing the relative weights of individual criteria, such as when specificity was originally given a weight of VH but later reduced to H, does not affect the final order of the rankings significantly. The only slight change is between [^11^C]PBB3 and [^18^F]THK5351. The initially integrated complete ranking did not shift, despite shifts in the outranking net flow values, positive net flow values, and negative net flow values. 

### 4.2. Comparison with Other Multiple-Criteria Decision Methods to Further Validate Our Approach 

We used the same set of criteria and weights as in the PROMETHEE method for the weighted sum and TOPSIS methods. The results obtained using the three methods are presented in Table 6.

## 5. Discussion

This study used a fuzzy PROMETHEE method to evaluate different tau PET radiotracers based on selected criteria, weights, and preference functions. The results showed that the [^18^F]RO-948 tau radiotracer was the preferred alternative, followed by [^11^C]RO-643. However, the ranking could change depending on the selected criteria and weights assigned to each criterion.

The strengths and weaknesses of each alternative are depicted in Figure 1. The diagram also shows the relative importance of each criterion, which is proportional to the disparity between the positive and negative preference flows.

To validate the results, a sensitivity analysis was performed to determine how changing the weight of a single criterion would affect the final ranking [51]. In this case, the specificity criterion was selected, and its weight was reduced from VH to H. The results show that the final ranking remained almost the same, indicating that the results are robust and that the application of the fuzzy PROMHEE method to tau PET radiotracers is not highly dependent on small changes in the criteria’s weight.

### 5.1. Comparison with Previous Studies to Further Validate Our Approach

In addition to comparing our results to those from other multiple-criteria decision methods, we assessed our results with reference to several studies that directly and empirically compared different tau PET radiotracers using methods such as autoradiography and/or quantification of PET signal in animal models or humans. Such studies generally compare only a subset of available radiotracers, and the results are therefore not directly comparable to ours. Still, available studies such as that by Smith et al. [52] do support the results of our study, showing that RO-948 may be a preferred radiotracer for most purposes, with superior imaging properties and diagnostic performance. More generally, our results are in accord with improvements in 2nd generation compared to 1st generation tracers.

Overall, the comparison with previous studies suggests that our fuzzy PROMETHEE approach is a promising method for evaluating and ranking tau PET radiotracers based on selected criteria, weights, and preference functions. The sensitivity analysis and validation with other methods further validates the results and shows the robustness of the method. 

### 5.2. Limitations of This Study

In this study, we made the first attempt at evaluating and comparing different tau PET radiotracers by conducting a comprehensive and filtered literature search to identify and weigh criteria. However, the results of MCDM methods depend critically upon the identification of relevant criteria, assignment of criteria weights, and scoring of alternatives, and this is limited by the information available and can be subjective. This is a fundamental challenge with these types of methods. This Inherent subjectivity in identifying criteria, assigning weights, and scoring alternatives, as well as the limited information available concerning these new, mostly experimental tau PET radiotracers, emphasizes the need for more research in this field. Furthermore, since different tau PET radiotracers are used in ways that may not be fully understood or agreed upon due to the fact of conflicting results in the literature and the newness of this research field, there is uncertainty and a lack of consistency with the scoring of alternatives and assigning of criteria weights. For this study, we included only a small subset of criteria known to be important for radiotracer usefulness, either because information about these criteria was not available for all tracers or because assigning scores for these criteria was too complex or too dependent upon specific contexts of use. For example, we did not include the following criteria: whether there were radiometabolites that would complicate PET quantification; whether a tracer is a substrate for blood–brain barrier efflux transporters that would affect net brain penetrance; whether a tracer is sensitive to 3R versus 4R tau, which would determine whether that tracer may be useful in diagnosing non-AD tauopathies; and estimates of whole-body or organ-specific radiation exposure. The implementation of different or supplementary criteria that may enhance or hinder the efficacy of tau radiotracers will be important in future studies. We also did not account for issues related to ^11^C versus ^18^F tracers: ^11^C tracers must be synthesized onsite and are therefore not appropriate for widespread or commercial use. However, ^11^C tracers involve less radiation exposure and allow more than one tracer to be administered at a single PET scanning session, since they decay quickly. 

Additional limitations include the need to reapply MCDM methodology to determine the outranking net flows in order to include other tau PET radiotracers in the evaluation and the lack of consideration of the variability introduced by equipment differences, including digital versus analogue PET equipment, which our methods cannot account for.

A key limitation is the lack of the clinical validation of the tau PET tracers evaluated in the study. While the study compared and ranked existing tracers based on selected criteria, the clinical effectiveness of these tracers is still being investigated and may vary depending on the specific disease and patient population. Further research could address these gaps by conducting more extensive clinical studies to validate the performance of the evaluated tracers and developing a systematic approach to weighting criteria in the decision-making process. Comparing, ranking, and choosing the best tau PET tracer for a particular purpose is a challenging and complex task that requires multidisciplinary expertise. The translation of MCDM tools into clinical settings as an effective decision aid tool requires more research and validation by clinical trials. Despite these limitations, the current results demonstrate, for the first time, the application of MCDM methods to the selection of a PET radiotracer. 

## 6. Conclusions and Recommendations

In this study, we used the fuzzy PROMETHEE decision-making model, an MCDM tool, to evaluate 15 different tau PET radiotracers used for the assessment of tau neurobrillary tangles in the brain. Tau PET is important for the diagnosis of tauopathies such as AD and for monitoring of treatment effectiveness. To the best of our knowledge, the fuzzy PROMETHEE methodology or any MCDM method has not been proposed for use in prioritizing promising and effective tau or any other PET tracers. We compared tau PET radiotracers and found that [^18^F]RO-948 performed best when used within the constrained system structure and requirements in terms of specificity, target binding affinity, brain uptake/penetration, and adverse effects. We obtained similar results when varying the criteria weights in a sensitivity analysis and when using two different MCDM methods. Our results also match available clinical studies that directly compare tau PET radiotracers. 

Further research can enhance this work by incorporating additional tau PET radiotracers when they become available, as well as using additional systematically selected criteria for decision making and evidence-based estimates of criteria weights and alternatives to selecting radiopharmaceutical for tau PET imaging. It will also be important that tau PET radiotracers be evaluated and compared using different combinations of decision-making models and algorithms and that outcomes from decision-making models be compared to clinical studies of radiotracer usefulness in different clinical populations and situations. This first application of MCDM methods in selecting a PET radiotracer demonstrates how such methods can help researchers and clinicians choose an appropriate tau PET radiotracer for specific purposes in order to advance research understanding and clinical care of neurodegenerative disorders.

## Figures and Tables

**Table 1:** Linguistic fuzzy scale of the assigned weights of importance to the criteria. VH: very high; H: high; M: medium; L: low; VL: very low.

Linguistic Scale	Triangular Fuzzy Scale	Criteria
Very High (VH)	(0.75, 1, 1)	Specificity, brain uptake, and penetration
High (H)	(0.50, 0.75, 1)	Target binding affinity
Medium (M)	(0.25, 0.50, 0.75)	Adverse reactions
Low (L)	(0, 0.25, 0.50)	
Very Low (VL)	(0, 0, 0.25)	

**Table 2:** Dataset for evaluating the tau PET radiotracers. VH: very high; H: high, M: medium; L: low; VL: very low.

Aim	Max.	Max.	Max.	Min.
Weight	VH	H	VH	M
Alternative/Criteria	Specificity	Target Binding Affinity	Brain Uptake and Penetration	Adverse Reactions
**First-generation tau PET radiotracers**				
[^18^F]AV-1451 [3,6,7,21,22,44,45]	VH	L	H	M
[^11^C]PBB3 [18,19]	H	M	H	M
[^18^F]THK5105 [7,8,21,22]	M	L	M	M
[^18^F]THK5117 [7,20,21,22]	M	L	M	M
[^18^F]THK5317 [7,7,21,22]	M	L	M	L
[^18^F]THK5351 [7,7,21,22]	H	L	M	L
**Second-generation tau PET radiotracers**				
[^18^F]MK-6240 [3,21,23,24,25]	H	VH	VH	L
[^18^F]GTP1 [23,46,47]	H	H	H	M
[^18^F]PM-PBB3 [48,49]	H	H	H	L
[^18^F]JNJ-067 [3,21,23,24,25]	H	VH	H	L
[^18^F]JNJ-311 [26]	H	H	H	L
[^11^C]RO-643 [32]	VH	VH	VH	M
[^11^C]RO-963 [32]	VH	VH	VH	M
[^18^F]RO-948 [3,21,23,24,25,32]	VH	VH	VH	L
[^18^F]PI-2620 [3,21,23,24,25,50]	H	VH	H	M

**Table 3:** Complete ranking of the tau PET radiotracers. Outranking net flow is the difference between positive and negative flows. The higher the outranking net flow, the more preferable the alternative.

Rank	Tau PET Radiotracers	Net Outranking Flow	Positive Outranking Flow	Negative Outranking Flow
1	[^18^F]RO-948	0.0051	0.0051	0.0000
2	[^11^C]RO-643	0.0045	0.0048	0.0003
2	[^11^C]RO-963	0.0045	0.0048	0.0003
4	[^18^F]MK-6240	0.0042	0.0043	0.0001
5	[^18^F]JNJ-067	0.0033	0.0036	0.0003
6	[^18^F]PI-2620	0.0027	0.0032	0.0006
7	[^18^F]PM-PBB3	0.0019	0.0023	0.0004
7	[^18^F]JNJ-311	0.0019	0.0023	0.0004
9	[^18^F]GTP1	0.0013	0.0020	0.0007
10	[^18^F]AV-1451	−0.0023	0.0014	0.0037
11	[^18^F]THK5351	−0.0041	0.0006	0.0048
12	[^11^C]PBB3	−0.0047	0.0003	0.0050
13	[^18^F]THK5317	−0.0057	0.0003	0.0060
14	[^18^F]THK5105	−0.0063	0.0000	0.0063
15	[^18^F]THK5117	−0.0063	0.0000	0.0063

**Table 4:** Selected importance weights of the criteria for the sensitivity analysis.

**Aim**	Max.	Max.	Max.	Min.
**Weight**	H	H	VH	M
**Alternative/Criteria**	Specificity	Target binding affinity	Brain uptake and penetration	Adverse reactions

**Table 5:** Sensitivity analysis results with fuzzy PROMETHEE.

Rank	Tau PET Radiotracers	Net Outranking Flow	Positive Outranking Flow	Negative Outranking Flow
1	[^18^F]RO-948	0.0049	0.0049	0.0000
2	[^11^C]RO-643	0.0043	0.0046	0.0003
2	[^11^C]RO-963	0.0043	0.0046	0.0003
4	[^18^F]MK-6240	0.0041	0.0043	0.0001
5	[^18^F]JNJ-067	0.0032	0.0035	0.0003
6	[^18^F]PI-2620	0.0026	0.0032	0.0006
7	[^18^F]PM-PBB3	0.0018	0.0023	0.0004
7	[^18^F]JNJ-311	0.0018	0.0023	0.0004
9	[^18^F]GTP1	0.0012	0.0020	0.0007
10	[^18^F]AV-1451	−0.0025	0.0012	0.0037
11	[^11^C]PBB3	−0.0033	0.0005	0.0038
12	[^18^F]THK5351	−0.0042	0.0005	0.0048
13	[^18^F]THK5317	−0.0057	0.0003	0.0060
14	[^18^F]THK5105	−0.0063	0.0000	0.0063
15	[^18^F]THK5117	−0.0063	0.0000	0.0063

**Table 6:** Comparison of the ranking results obtained using different methods. The results obtained with PROMETHEE, TOPSIS, and weighted sum methods are in very good agreement and differ only slightly.

Tau PET Radiotracers	PROMETHEE Net Flow	Rank (PROMETHEE)	Weighted Sum Score	Rank (Weighted Sum)	TOPSIS Score	Rank (TOPSIS)
[^18^F]RO-948	0.0051	1	0.0884	1	1.0000	1
[^11^C]RO-643	0.0045	2	0.0810	3	0.7684	3
[^11^C]RO-963	0.0045	2	0.0810	3	0.7684	3
[^18^F]MK-6240	0.0042	4	0.0838	2	0.8282	2
[^18^F]JNJ-067	0.0033	5	0.0792	5	0.7541	5
[^18^F]PI-2620	0.0027	6	0.0718	8	0.6646	8
[^18^F]PM-PBB3	0.0019	7	0.0748	6	0.6865	6
[^18^F]JNJ-311	0.0019	7	0.0748	6	0.6865	6
[^18^F]GTP1	0.0013	9	0.0671	9	0.5987	9
[^18^F]AV-1451	−0.0023	10	0.0588	10	0.4210	10
[^18^F]THK5351	−0.0041	11	0.0548	11	0.3209	12
[^11^C]PBB3	−0.0047	12	0.0543	12	0.3388	11
[^18^F]THK5317	−0.0057	13	0.0487	13	0.2316	13
[^18^F]THK5105	−0.0063	14	0.0408	14	0.0000	14
[^18^F]THK5117	−0.0063	14	0.0408	14	0.0000	14

## Data Availability

The data presented in this study are contained within the article.
